# Increased biomass production and glycogen accumulation in *apcE* gene deleted *Synechocystis* sp. PCC 6803

**DOI:** 10.1186/s13568-014-0017-z

**Published:** 2014-03-15

**Authors:** Ancy Joseph, Shimpei Aikawa, Kengo Sasaki, Fumio Matsuda, Tomohisa Hasunuma, Akihiko Kondo

**Affiliations:** 1 Department of Chemical Science and Engineering, Graduate School of Engineering, Kobe University, 1-1 Rokkodaicho, Nada-ku, Kobe 657-8501, Hyogo, Japan; 2 Core Research for Evolutional Science and Technology, Japan Science and Technology Agency, 3-5 Sanbancho, Chiyoda-ku 102-0075, Tokyo, Japan; 3 Organization of Advanced Science and Technology, Kobe University, 1-1 Rokkodaicho, Nada-ku, Kobe 657-8501, Hyogo, Japan; 4 Department of Bioinformatic Engineering, Graduate School of Information Science and Technology, Osaka University, 1-5 Yamadaoka, Suita 565-0871, Osaka, Japan; 5 RIKEN Biomass Engineering Program, 1-7-22 Suehirocho, Tsurumi, Yokohama 230-0045, Japan; 6 Precursory Research for Embryotic Science and Technology (PRESTO), Japan Science and Technology Agency, 3-5 Sanbancho, Chiyoda-ku 102-0075, Tokyo, Japan; 7 Department of Food Bioscience and Technology, College of Life Science and Biotechnology, Korea University, Seoul 136-713, Republic of Korea

**Keywords:** Cyanobacteria, Antenna truncation, Biomass production, Glycogen, Synechocystis sp. PCC 6803

## Abstract

The effect of phycobilisome antenna-truncation in the cyanobacterium *Synechocystis* sp. PCC 6803 on biomass production and glycogen accumulation have not yet been fully clarified. To investigate these effects here, the *apcE* gene, which encodes the anchor protein linking the phycobilisome to the thylakoid membrane, was deleted in a glucose tolerant strain of *Synechocystis* sp. PCC 6803. Biomass production of the *apcE*-deleted strain under photoautotrophic and atmospheric air conditions was 1.6 times higher than that of strain PCC 6803 (1.32 ± 0.01 versus 0.84 ± 0.07 g cell-dry weight L^−1^, respectively) after 15 days of cultivation. In addition, the glycogen content of the *apcE*-deleted strain (24.2 ± 0.7%) was also higher than that of strain PCC 6803 (11.1 ± 0.3%). Together, these results demonstrate that antenna truncation by deleting the *apcE* gene was effective for increasing biomass production and glycogen accumulation under photoautotrophic and atmospheric air conditions in *Synechocystis* sp. PCC 6803.

## Introduction

Biorefinery processes that convert sustainable biomass into commercially valuable chemical and energy sources have received increasing attention because of depleting fossil fuel reserves and concerns about the accumulation of greenhouse gases (Hasunuma et al. [2013a]). Recently, microalgae and cyanobacteria have been utilized as feedstocks for biorefinery processes, to produce third-generation fuels, due to their high photosynthetic efficiency compared with sugar or starch crops such as sugar cane and corn, and CO_2_-neutral fuel production (Schenk et al. [2008]; Nguyen et al. [2012]; Aikawa et al. [2013]). For example, bioethanol was produced directly from the cyanobacterium *Arthrospira platensis*, which contains high levels of storage glycogen, by a recombinant yeast strain diplaying an amylolytic enzyme (Aikawa et al. [2013]). To further increase the production of energy and valuable chemical products using these biorefinery processes, it is necessary to increase the mass productivity and carbohydrate content such as glycogen of oxygenic photosynthetic microorganisms.

At high light intensities, the rate of photon absorption by the chlorophyll antenna molecules in the surface layer of microalgal and cyanobacterial cells in cultures or pond environments exceeds the rate of photosynthetic reactions, resulting in the dissipation or loss of excess photons and/or photoinhibition of photosynthesis (Melis [2009]). In addition, as a result of this excess photon absorption by surface-layer cells, cells at greater distances from the surface are deprived of sufficient light to support photosynthesis (Melis [2009]). However, it was shown that a truncated chlorophyll antenna mutant of the green alga *Chlamydomonas reinhardtii* exhibited reduced absorbance of light by the first layers of cells and alleviated photoinhibition, resulting in high biomass production (Polle et al. [2003]).

The cyanobacterium *Synechocystis* sp. PCC 6803 has been widely studied as a model species for photosynthetic processes because of its transformability and full sequenced genome (Kaneko et al. [1996]). *Synechocystis* sp. PCC 6803 have a large membrane-extrinsic phycobilisome antenna, which is composed of rods of the pigment phycocyanin, an allophycocyanin core, and several linker proteins connecting the rods to the core and the core to the thylakoid membrane (MacColl [1998]). However, truncation of phycobilisome antenna by deleting phycocyanin rods or the entire phycobilisome assembly decreases biomass productivity under atmospheric air or 5% CO_2_ bubbling conditions (Page et al. [2012]). On the other hand, previous studies have shown that deletion of *apcE* gene, whose product is involved in the interection between the thylakoids and phycobilisome (Shen et al. [1993]), leads to the loss of phycobilins from the thylakoid fraction and decrease in antenna size (Shen et al. [1993]; Ajlani et al. [1995]). Deletion of *apcE* gene resulted in decreased biomass production under photoautotrophic and undefined CO_2_ conditions (Shen et al. [1993]). To date, therefore, the effect of *apcE* deletion on biomass production has not been fully investigated.

In this study, the effect of *apcE* gene deletion on the growth of *Synechocystis* sp. PCC 6803 was investigated under photoautotrophic conditions with atmospheric air, 1% CO_2_, or 2% CO_2_. In addition, the effect of *apcE* gene deletion on glycogen accumulation was also investigated. Atmospheric CO_2_ condition was effective to increase biomass production and glycogen accumulation in *apcE* gene deleted *Synechocystis* sp. PCC 6803.

## Materials and methods

### Microorganisms and growth condition

A glucose-tolerant strain of *Synechocystis* sp. PCC 6803 (here after referred to as GT) was obtained from Prof. Masahiko Ikeuchi of the University of Tokyo. *Synechocystis* sp. PCC 6803 was routinely cultured in BG11 medium, which contained 1.5 g L^−1^ NaNO_3_, 0.04 g L^−1^ K_2_HPO_4_, 0.075 g L^−1^ MgSO_4_ · 7H_2_O, 36 mg L^−1^ CaCl_2_ · 2H_2_O, 6 mg L^−1^ citric acid, 6 mg L^−1^ ferric ammonium citrate, 1 mg L^−1^ EDTA (disodium salt), 20 mg L^−1^ NaCO_3_, 2.86 mg L^−1^ H_3_BO_3_, 1.81 mg L^−1^ MnCl_2_ · 4H_2_O, 0.222 mg L^−1^ ZnSO_4_ · 7H_2_O, 0.39 mg L^−1^ NaMoO_4_ · 2H_2_O, 0.079 mg L^−1^ CuSO_4_ · 5H_2_O, and 49.4 μg L^−1^ Co(NO_3_)_2_ · 6H_2_O (Rippka [1988]), under continuous illumination at 50 or 200 μmol photons m^−2^ s^−1^ using white fluorescence bulbs (Life Look HGX and NHG; NEC, Tokyo, Japan) at 28 ± 2°C under atmospheric air conditions. For CO_2_ enriched cultivation, cells were cultivated at 50 μmol photons m^−2^ s^−1^ and 1 or 2% CO_2_ was supplied with a flow rate at 80 mL min^−1^. Light intensity was measured in the middle of the culture using an LI-250A light meter (LI-COR, Lincoln, NE) equipped with an LI-190SA quantum sensor (LI-COR). *Escherichia coli* strain DH5α was used to propagate pBluescriptSK- and the *apcE* inactivation plasmids.

### Cloning and transformation

For the construction of an *apcE* mutant, the 500-bp upstream and downstream regions of the *apcE* (slr0335) coding region of GT were isolated by PCR using the primers listed in Additional file 1: Table S1. The obtained DNA fragments were joined through standard PCR-driven overlap extension to form a single DNA segment harbouring a AatII restriction site in place of the coding region. After cloning the fused DNA fragment into pBluescriptSK, the resulting plasmids were digested with AatII, and an AatII-digested Km^r^ cassette, which was amplified from the pCR® II-Blunt-TOPO® vector (Toyobo Life science, Japan) was ligated into the plasmid in the same orientation as the coding region. The resulting deletion cassette was verified by PCR and nucleotide sequencing (Big Dye kit, ABI Perking Elmer). For the selection and maintenance of plasmids, the culture medium was supplemented with 50 μg L^−1^ of kanamycin. The vector containing the deletion casette was transformed into GT by homologous recombination and positive colonies were identified by PCR analysis. Selected clones were restreaked on plates supplemented with appropriate antibiotics to obtain complete deletion of the *apcE* gene. The *apcE* gene-disrupted GT mutant (∆*apcE* mutant) was routinely cultivated in BG11 medium supplemented with 50 μg L^−1^ of kanamycin under the same conditions used for GT, unless otherwise mentioned.

### Spectral analysis

After cultivation of GT for 9 days under the conditions described above, the culture was diluted with fresh medium to adjust the OD_750_ to 0.1. Steady-state absorption spectra were collected at room temperature using a spectrometer equipped with an integrating sphere (JASCO V-650/ISV-722), as described previously (Akimoto et al. [2013]). All absorption spectra were normalized to the chlorophyll Qy band (~676 nm).

### Analytical methods

Cell growth was monitored tubidimetrically by measuring OD_750_. Cell concentration in culture media is reported as dry cell weight, as a linear correlation was obtained between dry cell weight and optical density. It was determined that 1.0 OD_750_ equals approximately 0.26 g dry-cell weight L^−1^ both in GT and ∆*apcE* mutant.

The total carbohydrate content was analyzed by a colorimetric method using anthrone reagent (Updegraff [1969]). Briefly, 5 mg of algal powder was reacted in 75% sulfuric acid containing 2 g L^−1^ anthrone reagent for 15 min at 100°C. The absorbance of the resulting solution and glucose as standard was measured at 620 nm. Total protein content was extracted from 5 mg of cells as described previously (De Marsac and Houmard [1988]). Protein concentrations were determined using a Sigma QuantiPro BCA Assay Kit (Sigma-Aldrich, St Louis, MO, USA) as directed by the manufacturer’s protocol and using bovine serum albumin as a standard. Glycogen content was determined by high-performance liquid chromatography (HPLC) (Shimadzu, Kyoto, Japan) using a size exclusion HPLC column (OHpak SB-806 M HQ; Shodex, Tokyo, Japan) and a reflective index detector (RID-10A; Shimadzu) as described previously (Izumi et al. [2013]). Glycogen was extracted from the dried cells using a previously reported method with slight modifications (Ernst et al. [1984]). The cells used for the assay were collected by centrifugation at 6,300 × *g* for 2 min at 25 ± 2°C and washed once with 0.3 M ammonium carbonate, which was volatilized during the subsequent lyophilisation process.

Photosynthetic oxygen evolution by the cells was determined in the exponential growth phase with a Clark-type oxygen electrode (DW2/2, Hansatech Instruments Ltd., King’s Lynn, UK) controlled by a computerized oxygen monitoring system (OMS; Hansatech Instruments Ltd.). After system calibration, photosynthetic O_2_ evolution was monitored for 8 min at 30°C during irradiation at 50, 100, 200, 300, 600, 1200, 1800 μmol photons m^−2^ s^−1^ from a halogen light source. Cell concentration in the reaction mixture was adjusted to an OD_750_ of 1.0 with BG11 medium.

## Results

### Construction of an ∆*apcE* mutant

**Figure 1:**
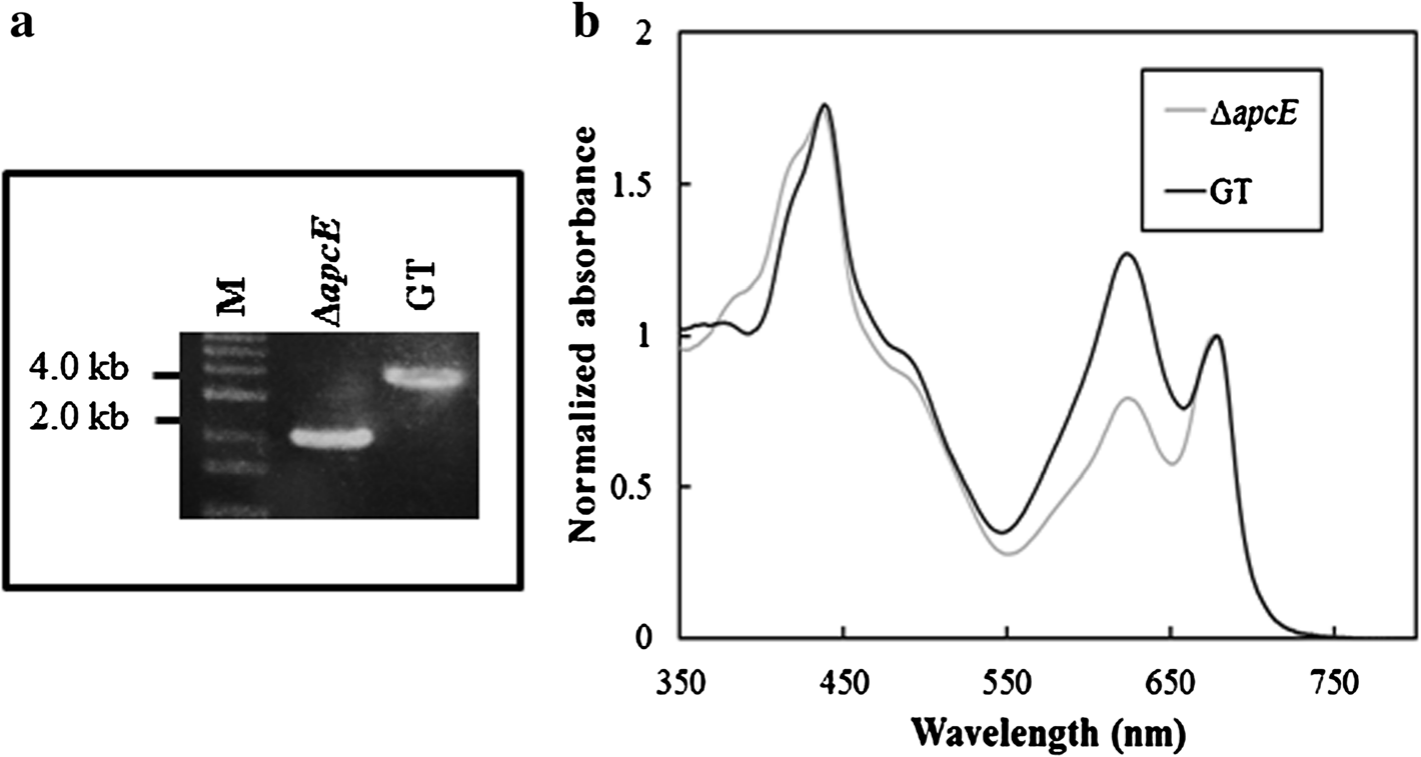
**Confirmation of complete deletion of the*apcE*gene. (a)** PCR with primers flanking the *apcE* gene for Δ*apcE* mutant (Lane 2) and glucose-tolerant *Synechocystis* sp. PCC 6803 (GT) (Lane 3). Lane 1 is DNA size marker (M). **(b)** Absorption spectral analysis of GT (black line) and Δ*apcE* (grey line).

Complete deletion of the *apcE* gene was confirmed by PCR analysis using the primers designed to bind 500 bp upstream and downstream of the *apcE* gene, as listed in Additional file 1: Table S1. The *∆apcE* mutant gave a single amplicon of 2.0 kb, confirming the deletion of the *apcE* gene and insertion of the kanamycin cassette, whereas GT produced an amplicon of 3.6 kb corresponding to the *apcE* gene (Figure 1a).

### Absorption spectra of GT and *∆apcE* mutant

For a quantitative analysis of the spectral features of GT and the *∆apcE* mutant, absorption spectra of intact cells were measured (Figure 1b). All absorption spectra were normalized at the chlorophyll Qy band. The absorption spectra of GT showed four peaks, which were assigned to the chlorophyll Soret (~435 nm), carotenoid (~500 nm), phycobilisome (~620 nm), and chlorophyll Qy (~676 nm) bands based on a previous report (Akimoto et al. [2013]). The *∆apcE* mutant showed a large reduction in the 620-nm absorption maximum of GT, as was previously observed for a phycobiliprotein-less mutant of *Synechocystis* sp. PCC 6803 (Shen et al. [1993]; Ajlani and Vernotte [1998]). The relative amount of chlorophyll (chlorophyll Soret and chlorophyll Qy) in the *∆apcE* mutant and wild-type GT were similar. These results suggested that deletion of the *apcE* gene, which would have impaired phycobilisome assembly and attachment, does not affect the amount of chlorophyll in cells.

### Effect of *apcE* gene deletion on growth of GT

**Figure 2:**
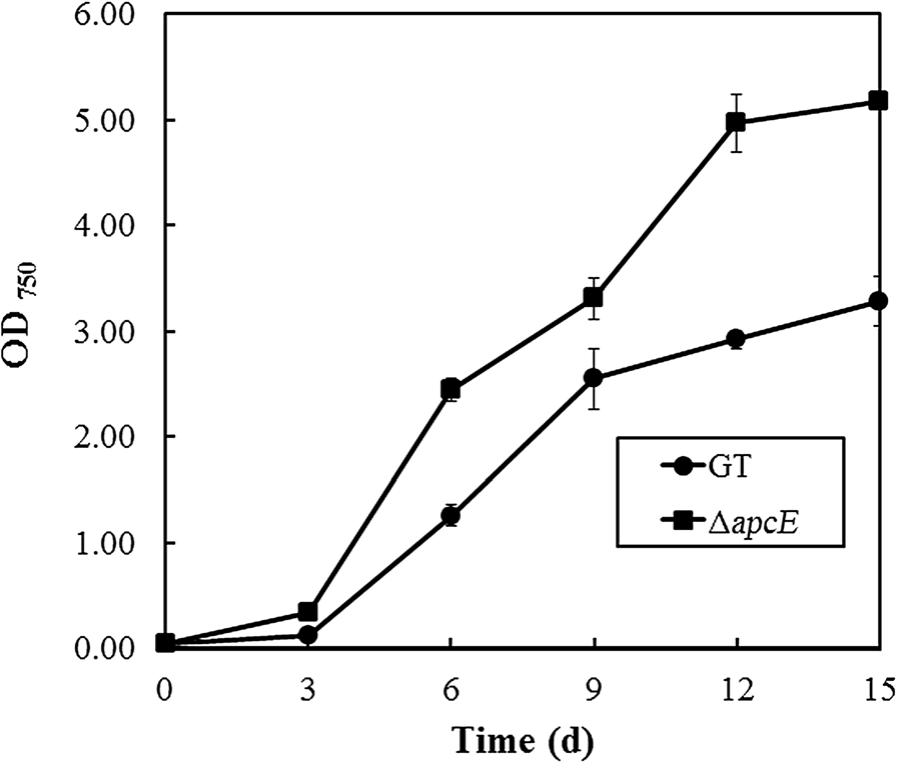
**Growth curves of glucose-tolerant*Synechocystis*sp. PCC 6803 (GT) (closed circles) and the Δ*apcE*mutant (closed squares) under photoautotrophic and atmospheric air conditions with illumination at 50 μmol photons m**^**−2**^ **s**^**−1**^**.** Error bars represent the mean of triplicate experiments.

**Figure 3:**
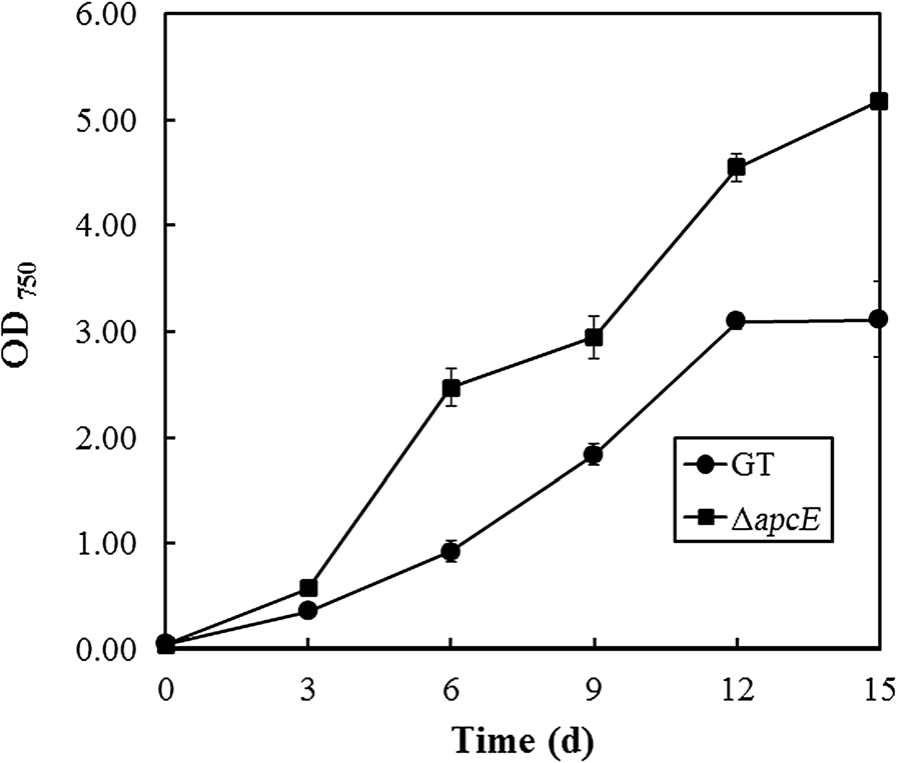
**Growth curves of glucose-tolerant*Synechocystis*sp. PCC 6803 (GT) (closed circles) and the Δ*apcE*mutant (closed squares) under photoautotrophic and atmospheric air conditions with illumination at 200 μmol photons m**^**−2**^ **s**^**−1**^**.** Error bars represent the mean of triplicate experiments.

**Figure 4:**
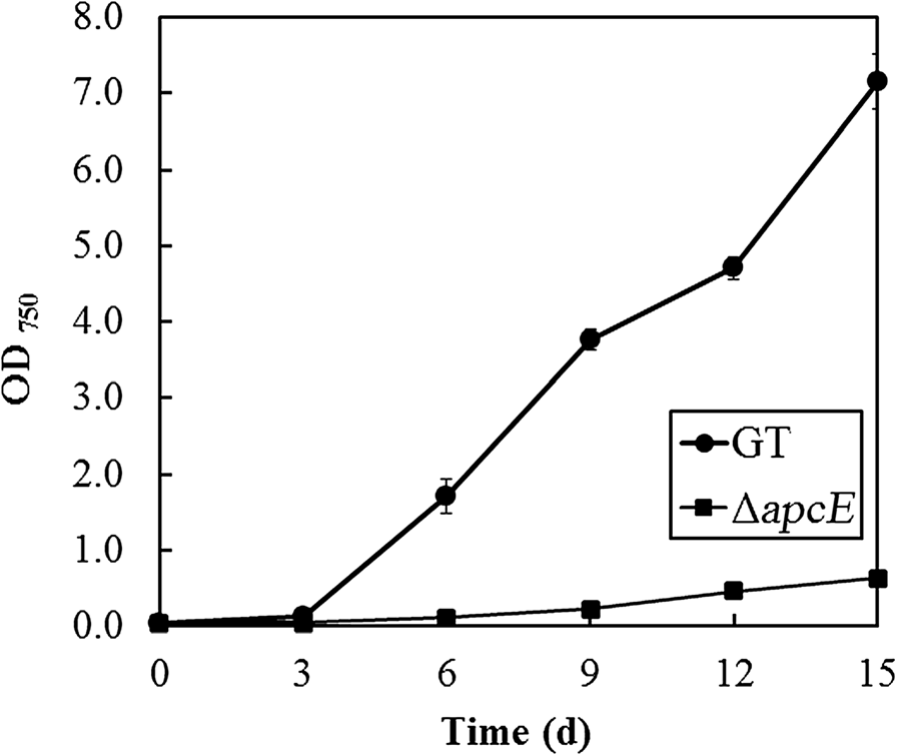
**Growth curves of glucose-tolerant*Synechocystis*sp. PCC 6803 (GT) (closed circles) and the Δ*apcE*mutant (closed squares) under photoautotrophic and 1% CO**_**2**_**conditions with illumination at 50 μmol photons m**^**−2**^ **s**^**−1**^**.** Error bars represent the mean of triplicate experiments.

To determine the impact of minimizing phycobilisome antenna size through deletion of the *apcE* gene on photoautotrophic growth, GT and the ∆*apcE* mutant were grown under atmospheric air conditions. Under these conditions, biomass production of the *∆apcE* mutant, as determined from OD_750_, was approximately 1.6-fold higher than that of GT after 15 days of cultivation with 50 μmol photons m^−2^ s^−1^ illumination (0.84 ± 0.07 and 1.32 ± 0.01 g-cell-dry weight L^−1^ for GT and the *∆apcE* mutant, respectively) (Figure 2). When the light intensity was increased to 200 μmol photons m^−2^ s^−1^ under atmospheric air condition, biomass production of the *∆apcE* mutant was higher than that of GT during 15 days of cultivation (Figure 3). However, under photoautotrophic (50 μmol photons m^−2^ s^−1^ illumination) and 1% CO_2_ condition, *∆apcE* mutant showed weak growth during 15 days of cultivation (Figure 4). In addition, the *∆apcE* mutant showed no growth by elevating CO_2_ to 2%, whereas the GT showed growth (data not shown). Deletion of the *apcE* gene caused a colour change as observed in the *∆apcE* mutant. The *∆apcE* mutant appeared olive green in colour due to a reduction in the amount of phycocyanin, as reported previously (Ajlani et al. [1995]), whereas GT cell appeared blue-green in colour.

### Cell compositions of GT and *∆apcE* mutant

To investigate the effect of *apcE* deletion on cell composition under photoautotrophic and atmospheric air conditions, carbohydrate and protein contents were analysed in GT and the *∆apcE* mutant after 15 days of cultivation with 50 μmol photons m^−2^ s^−1^ illumination (Table 1). The protein and carbohydrate contents in the *∆apcE* mutant were similar with those in GT, showing that *apcE* deletion did not affect protein and carbohydrate levels. However, in GT, only an average of 42.0 ± 0.3% of available carbohydrate was converted to glycogen, whereas the *∆apcE* mutant converted 85.0 ± 4.8% of carbohydrate to glycogen. Glycogen accumulation in the *∆apcE* mutant corresponded to approximately 24% of total dry cell weight after 15 days of cultivation, whereas the accumulated glycogen in GT cells corresponded to only 11% of dry cell weight (Table 1). At 200 μmol photons m^−2^ s^−1^ illumination and photoautotropic air condition, the glycogen content as a fraction of total dry cell weight was higher in the *∆apcE* mutant (25.8 ± 0.2%) than that of GT (18.5 ± 0.1%). These results showed that the restriction of photoantennae size directed carbon flux towards glycogen accumulation under photoautotrophic and atmospheric air conditions.

**Table 1:** **Biochemical composition of glucose-tolerant*Synechocystis*sp. PCC 6803 (GT) and the Δ*apcE*mutant under photoautotrophic and atmospheric air conditions with illumination at 50 μmol photons m**^**−2**^ **s**^**−1**^**on day 15**

**Strain**	**Dry-cell weight (g L**^**−1**^**)**	**Protein (% in dry-cell weight)**	**Carbohydrate (% in dry-cell weight)**	
			**Glycogen**	**Others**
GT	0.84 ± 0.07	47.3 ± 2.5	11.1 ± 0.3	15.4 ± 0.5
Δ*apcE*	1.32 ± 0.01	48.4 ± 0.2	24.2 ± 0.7	4.3 ± 1.5

Data are presented as the mean and standard deviation of three individual experiments.

### Effect of *apcE* gene deletion on photosynthesis

**Figure 5:**
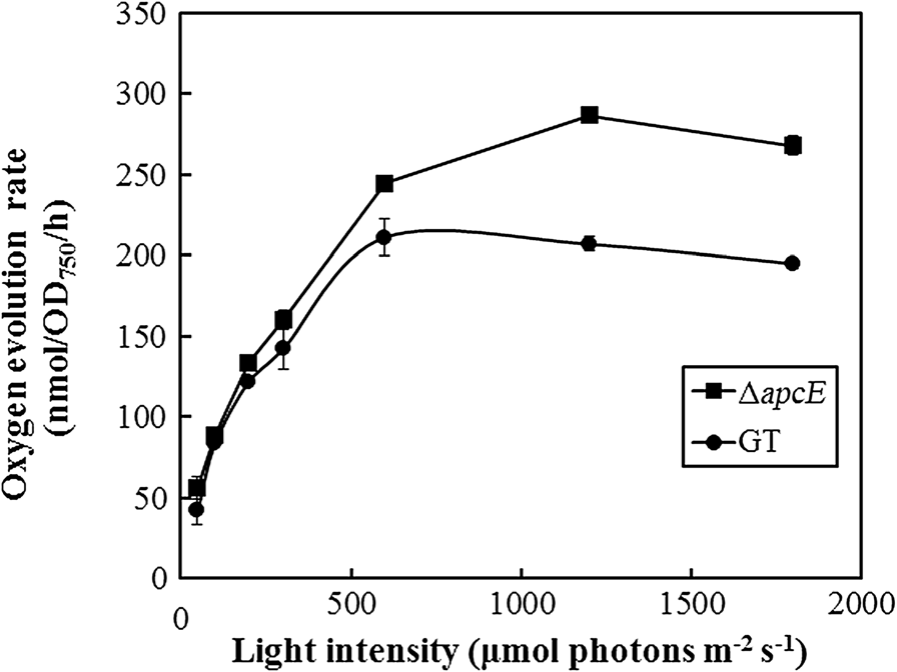
**Light-saturation curves for photosynthesis obtained for the Δ*apcE*mutant (closed squares) and glucose-tolerant*Synechocystis*sp. PCC 6803 (GT) (closed circles) under atmospheric air conditions.** Data are presented as the mean and standard deviation of three individual experiments.

The rate of oxygen evolution under *in*-*vivo* conditions with photoautotrophically grown cells of GT and the *∆apcE* mutant under different light intensities and atmospheric air condition was measured to generate light-photosynthesis curve (Figure 5). The rate of oxygen evolution by GT and *∆apcE* mutant cells at 50, 100, 200 and 300 μmol photons m^−2^ s^−1^ were similar, suggesting that the efficiency of photon use was similar by the two strains under these light conditions. However, the rate of oxygen evolution at 600, 1200, 1800 μmol photons m^−2^ s^−1^ was higher in the *∆apcE* mutant than that in GT, suggesting that the light-saturated evolution (*P*_max_) was higher in *∆apcE* mutant. This result was consistent with the previous finding that the green alga *Chlamydomonas reinhardtii* with a truncated chlorophyll antenna has an increased *P*_max_ (Polle et al. [2003]).

## Discussion

In this study, reducing the chlorophyll antenna size of the photosystems through mutation of the *apcE* gene in *Synechocystis* sp. PCC 6803 led to increased productivity, as determined by the increase in cell density under photoautotrophic and low CO_2_ conditions (0.04% in atmospheric air). The *∆apcE* mutant has a similar doubling time as the glucose-tolerant strain of *Synechocystis* sp. PCC 6803 (21.25 ± 0.55 h and 24.88 ± 0.81 h for GT and Δ*apcE* mutant, respectively*)*, between 3 to 6 days of culture. These doubling times are similar to that (25.2 h) reported previously for wild-type *Synechocystis* sp. cells (Page et al. [2012])*.* Deletion of the *∆apcE* gene increased the maximum cell density of cultures due to the decreased absorption of light by cells at the surface layer. Previous investigations by Nakajima and Ueda ([1997]) and Bernát et al. ([2009]) showed that the growth rate of a *Synechocystis ∆apcE* mutant was slower than that of the wild-type strain under photoautotrophic conditions with 1% CO_2,_ which corresponded with our results (Figure 4). Together, these findings suggest that the presence of high concentrations of CO_2_ might reduce the growth of *∆apcE* mutants. Although the reason behind this phenomenon is presently unclear, metabolic and gene expression analysis are expected to provide insight into this response and are currently underway in our laboratory. Under air conditions with illumination at 50 and 200 μmol photons m^−2^ s^−1^, the photosynthetic acitivities of the *∆apcE* mutant and wild-type strain were similar, as judged from the rate of oxygen evolution, although biomass production was higher in the *∆apcE* mutant, which was shown to markedly decreased levels of phycobilisomes. Thus, under photoautotrophic and low CO_2_ conditions, absorbed CO_2_ would be utilized by the *∆apcE* mutant for cell growth, rather than the production of phycobilisomes.

The accumulation of glycogen in cyanobacterial cells, which are attractive feedstocks for biorefinery processes (Aikawa et al. [2013]), was also increased in the *∆apcE* mutant compared with wild-type cells under photoautotrophic and atmospheric air conditions. In light conditions, cyanobacteria fix CO_2_ via the Calvin-Benson-Bassham (CBB) cycle, and CBB cycle intermediates enter central metabolic pathways, such as glycolysis and the tricarboxylic acid (TCA) cycle, and that leading to glycogen synthesis (Li and Liao [2013]). Glycogen synthesis is the major carbon and energy storage pathway, whereas glycolysis and the TCA cycle produce building blocks for cell growth. During prolonged nitrogen starvation, *Synechocystis* sp. PCC 6803 stores glycogen and degrades nitrogen-rich phycobilisomes, resulting in loss of the pigment phycocyanin, a condition which is referred to as bleaching or chlorosis (Krasikov et al. [2012]; Hasunuma et al. [2013b]). Here, deletion of the *apcE* gene caused a decrease in phycobilisomes, similar to observed in response to nitrogen starvation, and hence resulted in glycogen accumulation.

In conclusion, this is the first report that antenna truncation by *apcE* gene deletion in *Synechocystis* sp. PCC 6803 increased both biomass production and glycogen content under photoautotrophic and atmospheric air conditions. Due to these properties, this mutant is a potentially useful candidate for use in various biorefinery processes.

## Competing interests

The authors declare that they have no competing interests.

## Additional file

## Supplementary Material

Additional file 1: Primers used in this study. Click here for file
