# Supplementary material for: Increased biomass production and glycogen accumulation in apcE gene deleted Synechocystis sp. PCC 6803
Source: AMB Express. 2014 Mar 15;4:17. doi: 10.1186/s13568-014-0017-z (PMC4052703; doi:10.1186/s13568-014-0017-z)
Supplement: Additional file 1: — Primers used in this study. [file s13568-014-0017-z-S1.pdf]

Supplementary Table S1. Primers used in this study.

Primer Name	Sequence (5’-3’)
ApcE_up-F	CGGGCCCCCCCTCGAGGGGCATTTCCCACCCCGTTTCA
ApcE_up-R	ATGAATTGTTTTAGGACGTCTTGGATTTCATTATCTCCCATTAACA
ApcE_down-F	CTCATTTTAGCCATGACGTCGGTTGCGGTTGTTTAAAGCTTAGC
ApcE_down-R	GTGGATCCCCCGGGCTGCAGTGGGCATAATCCTCCAATTGGGCTAGC
kanamycin-F	ATAATGAAATCCAAGACGTCCTAAAACAATTCATCCAGTAA
kanamycin-R	AAACAACCGCAACCGACGTCATGGCTAAAATGAGAATATCACCG
ApcE gene-F	ATGAGTGTTAAGGCAAGTGG TGGC
ApcE gene-R	CTAACCGCCCACTTTTACTACTGGGGTA

Title: Increased biomass production and glycogen accumulation in *apcE* gene deleted *Synechocystis* sp. PCC 6803

Journal name: AMB express

Authors: Ancy Joseph, Shimpei Aikawa, Kengo Sasaki, Fumio Matsuda, Tomohisa Hasunuma, and Akihiko Kondo

Corresponding author: Akihiko Kondo

Affiliation: Department of Chemical Science and Engineering, Graduate School of Engineering, Kobe University

E-mail: akondo@kobe-u.ac.jp
